# Automatic text classification of prostate cancer malignancy scores in radiology reports using NLP models

**DOI:** 10.1007/s11517-024-03131-x

**Published:** 2024-06-07

**Authors:** Jaime Collado-Montañez, Pilar López-Úbeda, Mariia Chizhikova, M. Carlos Díaz-Galiano, L. Alfonso Ureña-López, Teodoro Martín-Noguerol, Antonio Luna, M. Teresa Martín-Valdivia

**Affiliations:** 1 Department of Computer Science, Advanced Studies Center in ICT (CEATIC), Universidad de Jaén, Campus Las Lagunillas, Jaén, 23071 Spain; 2 Natural Language Processing Unit, HT Médica, Carmelo Torres, no̱2, Jaén, 23007 Spain; 3 MRI Unit, Radiology Department, HT Médica, Carmelo Torres, no̱2, Jaén, 23007 Spain

**Keywords:** PI-RADS classification, Natural language processing, Radiology report classification, RoBERTa-clinical, XGBoost

## Abstract

**Abstract:**

This paper presents the implementation of two automated text classification systems for prostate cancer findings based on the PI-RADS criteria. Specifically, a traditional machine learning model using XGBoost and a language model-based approach using RoBERTa were employed. The study focused on Spanish-language radiological MRI prostate reports, which has not been explored before. The results demonstrate that the RoBERTa model outperforms the XGBoost model, although both achieve promising results. Furthermore, the best-performing system was integrated into the radiological company’s information systems as an API, operating in a real-world environment.

**Graphical abstract:**

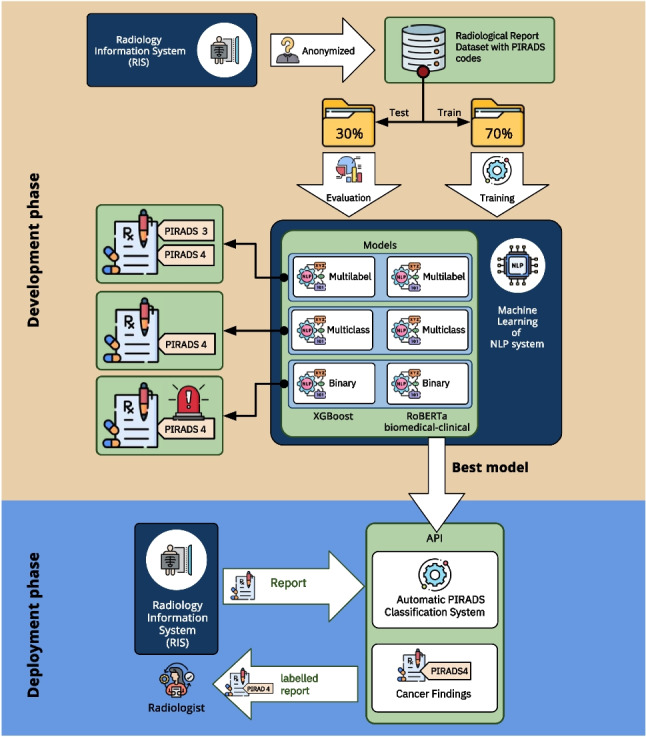

## Introduction

The application of Artificial Intelligence (AI) in the field of medicine, and specifically Natural Language Processing (NLP), is emerging as a highly beneficial approach for enhancing decision-making within medical information systems [1]. NLP is used to process large amounts of unstructured clinical data, such as medical progress notes and patient admission records, to identify patterns and relationships that may not be evident to physicians when reviewing reports manually.

In healthcare, the abundance of unstructured information, like medical notes and reports, necessitates swift processing and analysis. This capability significantly enhances healthcare efficiency and facilitates informed decision-making. For instance, NLP aids in identifying patterns and risk factors, facilitating early detection of diseases like prostate cancer.

Early detection of prostate cancer is essential for successful treatment and prevention of serious complications. Several methods and techniques are being applied to accomplish this early detection. One such method is prostate MRI (Magnetic Resonance Imaging), a non-invasive imaging technique that has become increasingly popular for the early detection of prostate cancer. The Prostate Imaging Reporting and Data Reporting System (PI-RADS) is a tool that has been developed to standardize the interpretation of prostate MRI images by ranking the imaging findings on a scale of 1 to 5, with higher scores indicating a higher risk of prostate cancer [2]. The PI-RADS score is used to assess the risk of prostate cancer in patients with suspicious prostate lesions at MRI studies and can help physicians determine the course of treatment and management options for prostate cancer patients.

Radiologists may have different levels of experience and skill in interpreting PI-RADS images, which may affect the accuracy of the assessment. In addition, the manual process can be error-prone, time-consuming and resource-intensive.

Automated detection of PI-RADS codes using NLP techniques is a promising solution that can be used to be more efficient and faster in detecting prostate cancer. NLP can analyze prostate image reports and automatically detect the associated PI-RADS code. This can improve the accuracy and efficiency of PI-RADS assessment, as manual assessment can be subjective and vary between radiologists. Automatic detection of PI-RADS codes can also reduce the time required for image assessment, which can improve the efficiency of the healthcare process and allow for more informed decision-making.

In this study, we present a novel approach employing advanced NLP techniques and Machine Learning (ML) algorithms for the development of a robust and automated system capable of accurately determining the PI-RADS code from radiological reports. Our proposed system aims to not only improve the accuracy of the PI-RADS evaluation but also reduce the time and effort required to conduct manual evaluations, making it a valuable tool for both experienced radiologists and novice practitioners who may not be as familiar with the PI-RADS classification system. To the best of our knowledge, this is the first time a system is being proposed to automatically detect PI-RADS codes using NLP techniques on Spanish reports. Additionally, we will employ two approaches: one based on traditional machine learning and another using the new transformers, demonstrating that the latter option outperforms the results obtained with traditional learning.

To conduct our research, we employ a corpus of radiological reports written in Spanish provided by the medical company HTmédica. This high-quality corpus consists of 5,000 reports that have been manually annotated by expert radiologists specialized in prostate cancer screening and labeled with one or more PI-RADS codes. With this corpus, we aim to develop and evaluate multiple approaches to ultimately implement a system that can assist radiologists in the accurate classification of PI-RADS codes. By recommending the most appropriate code for each case, our system aims to provide valuable support to radiologists, enhancing the efficiency and accuracy of prostate cancer screening.

The main contributions of our study can be summarized as follows:

- Developing several models for classifying PI-RADS categories using NLP techniques.
- Comparing the performance of different models and selecting the best one based on performance, efficiency, and other criteria.
- Implementing the models as an Application Programming Interface (API) that can be easily integrated into real clinical environments.
- Evaluating the model’s performance in a real-world environment and demonstrating its potential to improve clinical decision-making and patient outcomes.

## Related work

The radiology report is one of the main means of communication between physicians and patients using not only images but also free text to include the findings found in the radiological images. This fact makes radiology particularly suitable to benefit from the application of NLP [3]. Recently, many approaches have been developed to take advantage of the information in free-text radiological reports. Among the most common applications, we can highlight information extraction systems that aim to detect specific clinically relevant terms within the context of the reports [4, 5]; clinical question answering systems [6]; applications that generate summaries [7]; textual classification based on radiology reports [8] and even fine-grained sentiment analysis that extract a physician’s opinion of a patient’s health status [9].

In the field of radiology, automatic coding and classification systems encompass various computer-based approaches that assign standard terminology codes to clinical reports using NLP. For example, the ICD-10 (International Classification of Diseases, 10th revision) coding system extensively used in medicine allows the classification of a wide range of medical diagnoses and procedures covering diseases, disorders, injuries, symptoms, risk factors and external causes of disease. Thus, [10] demonstrated how applying NLP in ICD-10-based coding can improve the efficiency and accuracy of radiology reporting. These coding systems can structure relevant information, and improve patient detection, localization, characterization, and risk stratification.

**Fig. 1:**
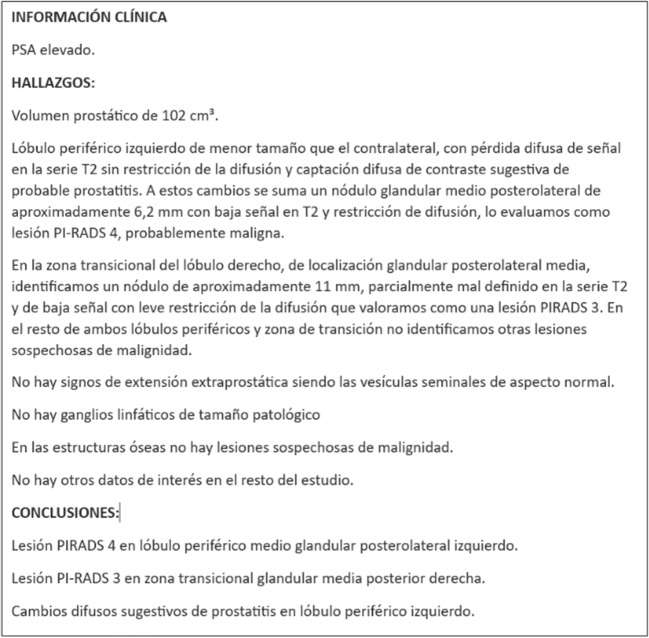
The sample of medical text reports extracted from the dataset

Recently, PI-RADS scoring has started to attract more attention from the scientific community which led to the development of different types of systems aimed to relieve the burden of PI-RADS v2.1 code assignment from radiologists. Given that a radiology report constitutes an interpretation of an imaging exploration, many automatic PI-RADS coding systems focus on leveraging images. For instance, [11] introduced a deep learning computer vision model that was designed to assist the radiologist in PI-RADS scoring of a patient’s lesion. Bijl et al. [12] implemented a system based on Convolutional Neural Networks (CNN) and image Transformer networks that perform semantic image segmentation, lesion detection and classification to assign PI-RADS class to pathologies.

As far as we are aware, not much work has been focused on utilizing NLP methods for assigning PI-RADS codes to radiology reports, particularly when dealing with free-text reports. In this context, [13] propose a rule-based algorithm to categorize prostate mpMRI (Multiparametric MRI) reports according to PI-RADS. Nevertheless, rule-based methods present challenges when adapting to different languages or institutions due to variations in language and reporting practices. Moreover, the time-consuming process of manually creating intricate rule sets for assigning PI-RADS codes to reports requires extensive domain knowledge, attention to detail, and poses difficulties in system maintenance and updates as language and clinical practices evolve.

Regarding ML research, [14] propose a decision tree classifier that takes as input a term frequency vector of the relevant n-grams detected in reports after regular expression-based filtering. This system is reported to show high Precision for relatively low-risk classes (Precision of PI-RADS 3 and PI-RADS 2 reaches 91.4% and 84.4%, respectively). The overall weighted average F1-score for the decision tree classifier reaches 69.9%.

Notably, prior research lacks focus on the automatic classification of radiological reports using PI-RADS v2.1 for Spanish-written reports or advanced NLP techniques based on language models and ML. Addressing this gap, our study utilizes a Spanish dataset of radiology reports labeled with PI-RADS categories. We aim to train and evaluate two classification systems: one employing traditional ML and the other based on language models.

## Dataset

In this study, we assembled a corpus of 5,000 unstructured Spanish radiological MRI reports spanning from April 2019 to June 2022, each annotated with PI-RADS categories. The reports were obtained from the HTmédica Radiology Information System (RIS) database, mainly comprising multiparametric prostate scans (49.8%) and pelvic viscera examinations (38.8%).

**Table 1:** Distribution of PI-RADS categories in the training and test sets

	PI-RADS 1	PI-RADS 2	PI-RADS 3	PI-RADS 4	PI-RADS 5
Training set	1,107	1,945	697	760	391
Test set	475	834	298	326	168

An example report displayed in Fig. 1 showcases two suspicious prostate lesions, each assigned different PI-RADS categories (PI-RADS 3 and PI-RADS 4). The reports generally consist of three sections: clinical information, findings, and conclusions, with an average length of 132 tokens and a standard deviation of 36.9 tokens. The reports were de-identified, removing patient and specialist details. The longest report reaches 511 tokens and the shortest is 72-token long.

**Fig. 2:**
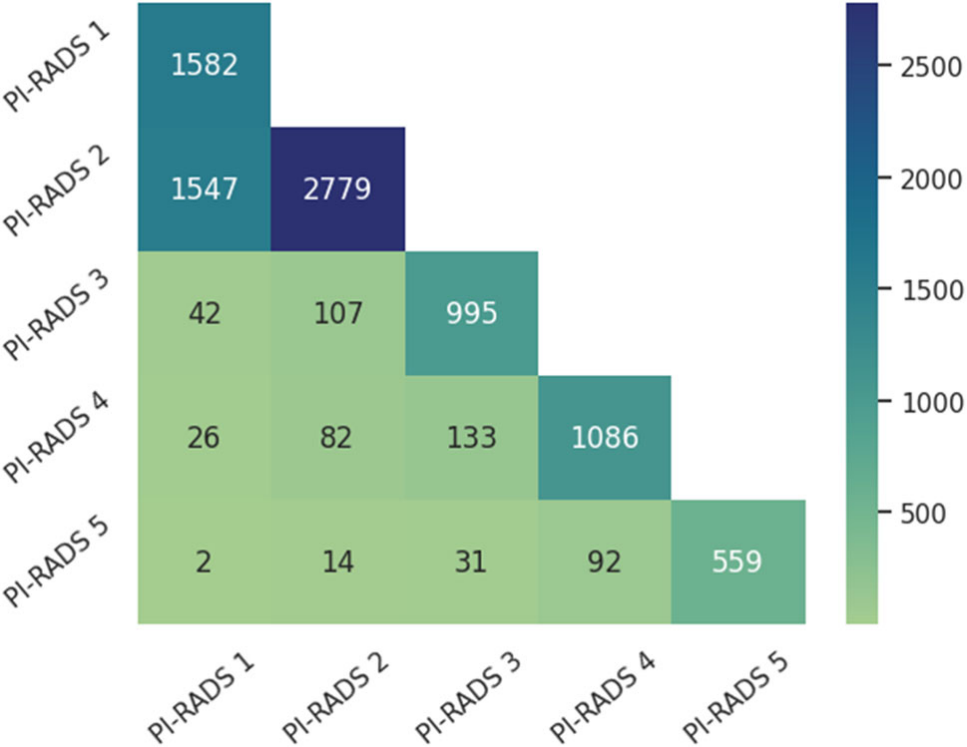
Label co-occurrence matrix in the annotated dataset

Each report was meticulously labeled by a radiologist with fourteen years of experience, considering the PI-RADS v2.1 categories. As multiple lesions could be present within a single report, it is a multilabel problem where a report might have more than one PI-RADS code. The distribution of labels varies, with most documents (3,074) containing a single label, 1,851 reports with 2 PI-RADS codes, and 75 reports including 3 different categories. The most frequent co-occurring codes in reports with multiple annotations are PI-RADS 1 and 2, while combinations of high-risk codes (4 or 5) with lower-risk ones (1 or 2) are less common, as shown in Fig. 2.

We have utilized 3,514 reports (70% of the total) for training and refining the automatic classification systems, while the remaining 1,486 reports (30% of the total) were selected and held out as an independent test set for the final evaluation. To ensure an unbiased dataset, we employed a stratified approach. The class distributions for both datasets are presented in Table 1.

Radiologists often include the PI-RADS classification in some of the prostate reports. More specifically, 3,026 reports (60% of the total) contained at least one explicit textual mention of the PI-RADS code assigned. In order not to bias the algorithms with these key terms we decided to remove them including variations like PI-RADS 3, PR2, P-Rads 5, etc.

## Methods

**Fig. 3:**
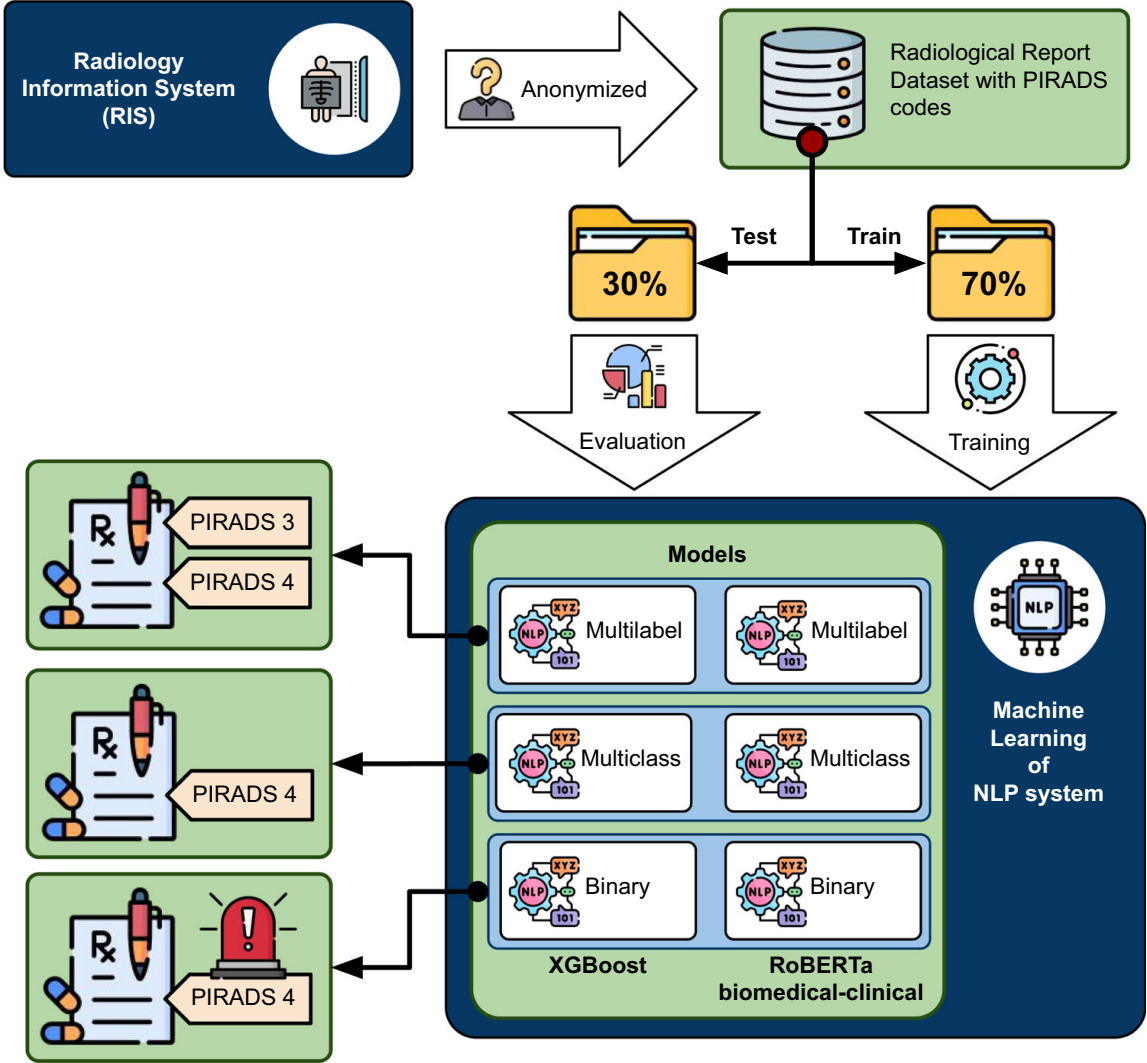
Dataset generation and model training process

In this section, we outline the machine learning (ML) methods employed—XGBoost and RoBERTa-clinical—for solving the automatic classification of PI-RADS codes in radiology reports using three different approaches: multilabel, multiclass, and binary classification. The overall process of dataset creation, partitioning, ML model usage, and classification types is summarized in Fig. 3.

### Machine learning approaches

Two different ML approaches have been tested, one based on traditional ML using gradient boosting (XGBoost) and another based on language models (RoBERTa-clinical) [15].

**XGBoost** In this approach, each text has been vectorized using the TF-IDF technique, where the vectorizer is first adjusted to the training set, and then used to transform all texts into TF-IDF features. Subsequently, the XGBoost algorithm [16] is trained with the vectorized texts from the training set and used to generate predictions on the test set. XGBoost is a popular and efficient open-source implementation of the gradient boosting trees algorithm. It is an ensemble learning method that combines the predictions of multiple weak models to produce a stronger prediction.

**RoBERTa-clinical** The transformer-based model chosen for this approach is RoBERTa biomedical-clinical, which is a model based on RoBERTa pre-trained with a combination of biomedical and clinical corpora in Spanish [17]. To fine-tune this model we have used the training set and HuggingFace’s transformer library [18] and adjusted its hyperparameters to maximize the macro F1 metric in the test set. This optimization has been performed thanks to the Optuna framework [19], which looks for a good combination of hyperparameters, also known as a trial, amongst a predefined search space. The range of values defined for this search space is detailed below:

- Learning rate: float value between 3e-5 and 5e-5.
- Training batch size: 8, 16, and 32.
- Evaluation batch size: 8, 16, and 32.
- Weight decay: float value between 1e-12 and 1e-1.
- Warmup steps: integer value between 0 and 1000.
- Random seed: integer value between 320 and 327.

To avoid overfitting on the training set, we halted the training process when the reference metric (macro F1) did not improve for 10 consecutive epochs by using an early stopping mechanism.

### Classification problem approaches

In our experimentation, we will solve the classification problem using three different approaches, from higher to lower complexity since we will deal with the problem of multilabel, multiclass, and binary classification.

#### Multilabel classification

A multilabel classification problem refers to a task in ML and data analysis where an instance or document (in our case, a radiology report) is associated with multiple labels simultaneously. Thus, instead of assigning a single label to a document, the goal is to predict multiple labels that are relevant to the document. The number of labels associated with each document can vary, ranging from zero to multiple labels. The objective is to develop a model that can accurately predict the appropriate labels for unseen instances based on the patterns and relationships learned from labeled training data.

The original dataset we are working on contains reports tagged with several tags in the same report. Thus, in the most complex case, models (XGboost and RoBERTa-clinical) have been trained to solve this multilabel classification problem. It is important to note that the first algorithm is not able to generate multilabel outputs natively, thus, it is necessary to use Sklearn’s MultiOutputClassifier tool [20], which automatically fits a classifier for each target class. Regarding the transformer approach, a custom loss function that combines a sigmoid layer with binary cross entropy is used to tackle this task.

#### Multiclass classification

In a multiclass classification problem the instances are categorized into one of three or more mutually exclusive classes or categories. Thus, the goal is to assign a single label to each instance from a set of possible labels.

**Table 2:** Performance metrics over the evaluation set

Task	Model	Macro-avg		
		Precision	Recall	F1-score
Multilabel	**XGBoost**	0.9415	0.8884	0.9140
	**RoBERTa-clinical**	0.9689	0.9500	0.9593
Multiclass	**XGBoost**	0.8862	0.8747	0.8799
	**RoBERTa-clinical**	0.9685	0.9340	0.9485
Binary	**XGBoost**	0.9630	0.9569	0.9598
	**RoBERTa-clinical**	0.9839	0.9816	0.9827

In multiclass document classification, each document is assigned to only one class out of the available classes (PI-RADS 1, PI-RADS 2, PI-RADS 3, PI-RADS 4, PI-RADS 5). The objective is to develop a model or algorithm that can accurately classify unseen documents into their appropriate classes based on patterns and relationships learned from labeled training data.

To tackle this task we first processed the original golden labels from the multilabel approach to adapt them for a multiclass classification. We made this by keeping the index of the highest-risk positive label for each report as the target for the multiclass problem. For example, a report containing the labels PI-RADS 2 and PI-RADS 3 is only labeled with the PI-RADS 3 class.

Regarding the training of the models, the ML approach no longer needs to fit one classifier for each target as the XGBoost algorithm can perform multiclass classification natively. Concerning the transformer approach (RoBERTa-clinical), the main difference with the previous task is related to the loss function, which now computes the cross entropy loss between the input logits and the labels. The search space used for the hyperparameter optimization remains the same as in the previous task.

#### Binary classification

In the binary classification problem, the instances are classified into one of two mutually exclusive classes or categories. The goal is to assign a single label to each instance, indicating whether it belongs to one class (positive class) or the other (negative class).

**Fig. 4:**
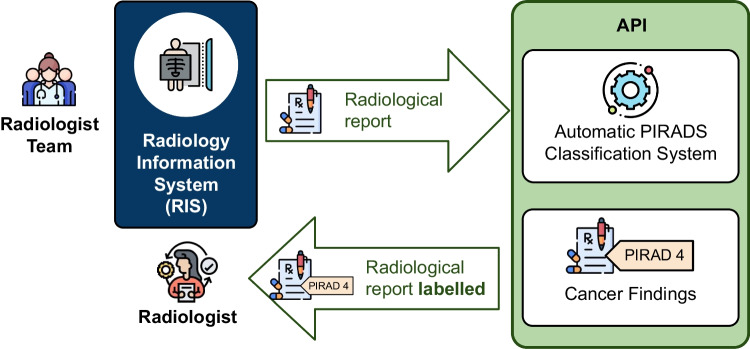
Diagram of the API integration into clinical practice

In terms of complexity, binary classification is generally considered less complex than multiclass or multilabel classification problems. This is because binary classification involves distinguishing between only two classes, which simplifies the decision-making process for the model. The model learns to differentiate instances based on a single boundary or threshold, making it relatively easier to train and evaluate.

In our case, we will adapt the dataset to consider reports labeled with PI-RADS 1, PI-RADS 2, and PI-RADS 3 as the negative class, while reports labeled with PI-RADS 4 and PI-RADS 5 will be considered the positive class. Thus, we can treat the problem as a case of binary classification. The reason we have made this corpus adaptation is that in a real-world setting, expert radiologists consider that PI-RADS codes 4 and 5 should be considered high risk, so we annotate reports containing one of these codes in the multilabel targets as the positive class to develop a binary classification model that learns to distinguish between high-risk and low-risk reports.

As binary classification can be seen as a sub-type of multiclass classification where the number of labels is two, the whole experimental setup for this problem is the same as in the previous one.

## Experimental results

In this section, we present the results obtained by all the systems developed for each classification task (see Table 2).

The RoBERTa-clinical system achieves the best results in all metrics (precision, recall and F1-score). Nevertheless, the results obtained by both systems have been very high. The difference in the Macro F1 measure for the multilabel classification task is 0.045 points (0.9593 for RoBERTa-clinical and 0.9140 for XGBoost). In the multiclass labeling task the difference between the two models is larger (about 0.07 points). The best results (0.9598 for XGBoost and 0.9827 for RoBERTa-clinical) and the smallest difference (0.023 points) between the two systems are obtained in the binary classification task.

## API integration and the clinical impact

Finally, we have developed an API that allows the prediction of new reports from the selected model in a real environment (see Fig. 4). The classification model selected for the API implementation has been the multiclass system as it is the most operational for radiologists. In addition, the RoBERTa-based model has been selected for the implementation of the API as it is the one with the best results and, although the training of these models is harder, once the system is trained, the production mode is very fast and effective.

To evaluate the performance of the system in a real-world environment, an expert radiologist annotated the first 350 reports of prostate explorations that were performed in the clinical company after the integration of the API into RIS. This allowed us to conduct experiments with data that was not used directly (as training input) and indirectly (as validation data for hyperparameter optimization) to adjust the model’s weights. Table 3 shows the metrics computed during this evaluation and Table 4 discloses metrics’ values per class.

**Table 3:** Averaged performance metrics over the API’s output

Task	Model	Macro-avg		
		Precision	Recall	F1-score
Multiclass	**RoBERTa-clinical**	0.7354	0.6642	0.6747

In terms of precision, which measures the proportion of true positives out of the total of instances, the classifier shows a high value mainly for the imbalanced label, PI-RADS 2. The detection of PI-RADS 3 results in the highest number of false positives all of them implying confusion with PI-RADS 4. For PI-RADS 2, the recall is also high at 0.9504, however, for PI-RADS 1 it is 0.333, probably because of the lack of data for that category. In terms of F1-score, the classifier achieves relatively high scores for PI-RADS 2, 4, and 5, ranging from 0.6796 to 0.9705, but shows lower values for PI-RADS 3 and 1: 0.4444 and 0.5484 respectively.

### Performance analysis

**Fig. 5:**
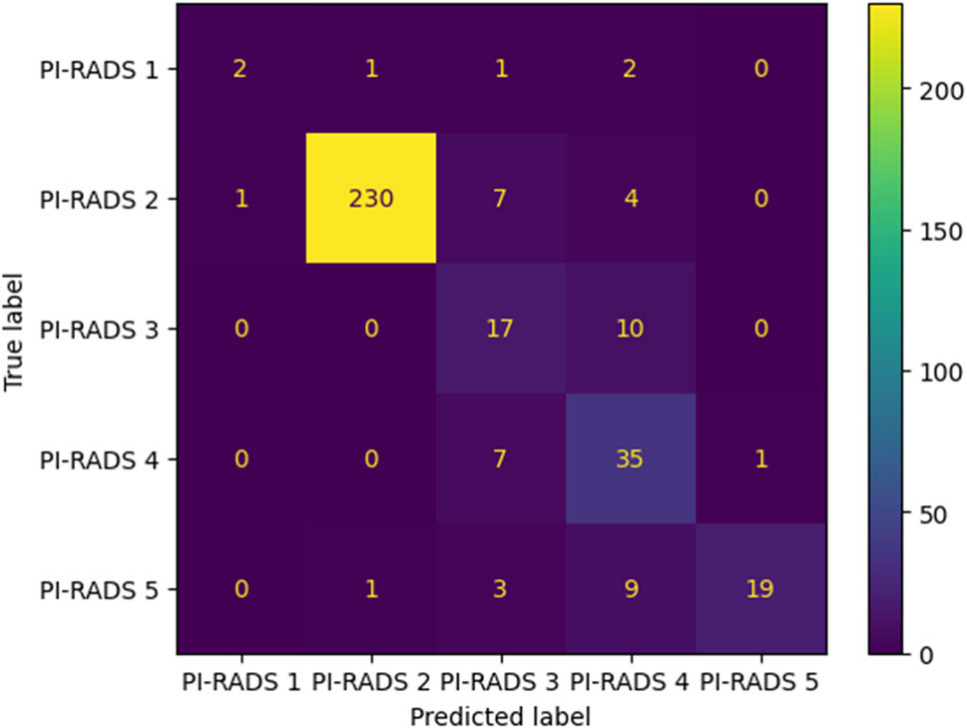
Confusion matrix for the API’s outputs

An in-depth analysis of errors encountered in predictions by the APIs was conducted on the evaluation corpus, comprising 350 reports, 47 of which were classified incorrectly. Notably, issues were primarily related to identifying PI-RADS 1 and 3. The confusion matrix in Fig. 5 highlights 5 and 10 false positives for these classes respectively.

**Table 4:** Metrics per class over the API’s output

Criteria	Precision	Recall	F1-score	Support
PI-RADS 1	0.6667	0.3333	0.4444	6
PI-RADS 2	0.9914	0.9504	0.9705	242
PI-RADS 3	0.4857	0.6296	0.5484	27
PI-RADS 4	0.5833	0.8140	0.6796	43
PI-RADS 5	0.9500	0.5938	0.7308	32

From the risk evaluation perspective, the errors are distributed equally with 21 cases of underestimation and 26 cases of overestimation. The cases of overestimation occurred mostly while processing reviews labeled with PI-RADS 2 and 3 by the expert radiologist. The underestimation happened mostly predicting PI-RADS 3 as PI-RADS 4 and PI-RADS 4 as PI-RADS 5.

**Fig. 6:**
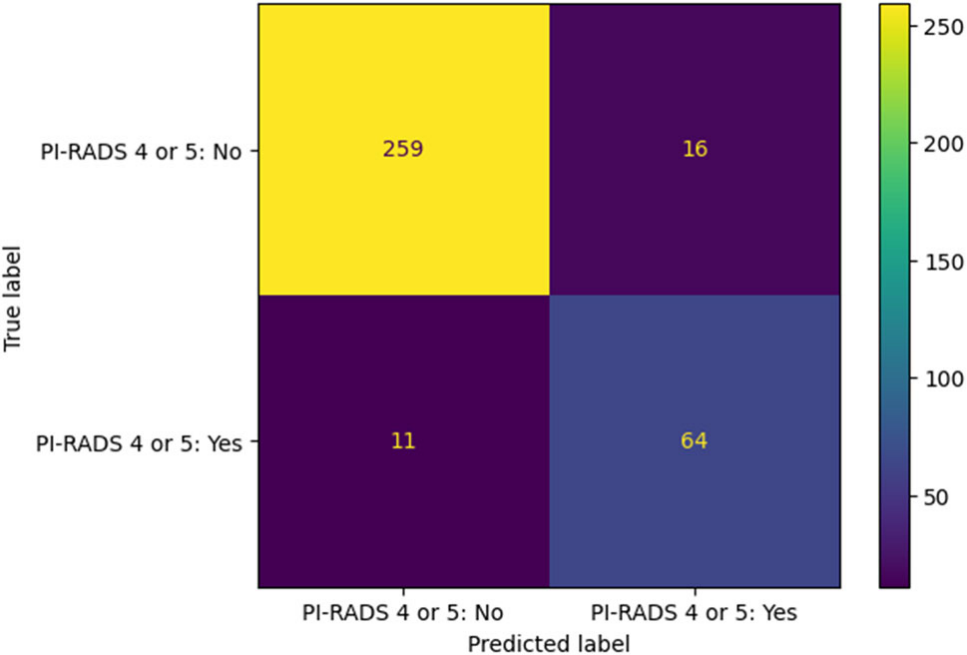
Pi-RADS 4 or 5: No or Yes. Binary confusion matrix for high-risk classes

Figure 6 shows the confusion matrix for distinguishing only between low and high-risk classes, i.e., Pi-RADS 1, 2, and 3 against Pi-RADS 4 and 5. This perspective highlights the system’s performance in detecting dangerous classes as it achieves a precision of 0.8000, a recall of 0.8533, and a F1-score of 0.8258.

### Clinical impact

Detection and notification of PI-RADS categories in radiology reports are mandatory as these categories, usually PI-RADS 4 and 5, encompass further actions such as prostate biopsy [21]. The goal of developing an automatic tool based on NLP for the detection of these relevant categories is focused on assisting radiologists in their common daily workflow. Once the radiology report is finished, these kinds of tools help the radiologist to ensure that those reports with relevant findings are correctly tagged and promptly sent to requesting physicians (or even patients). Regarding reporting tagging, the use of NLP tools based on the description of radiology findings on prostate MRI studies may help to standardize the way these categories are assigned. Moreover, this kind of NLP algorithm guides less experienced radiologists in the adoption of a lexicon related to PI-RADS classification. Concerning early notification of relevant findings, the huge workload that radiologists support every day, together with the continually increasing in MRI prostate studies since the last decade, led to a non-negligible, but almost inevitable punctual loss of communication of those relevant findings to urologists or oncologists [22]. This fact, linked with the current delay in specialists’ schedules may lead to a postponement of performing further actions regarding patients with clinically relevant PI-RADS categories, which may have a potential impact on their prognosis, therapeutic options, and outcome [23].

## Conclusions

This study presented two text classification systems for identifying prostate cancer findings based on PI-RADS codes: a traditional machine learning model (XGBoost) and a language model-based approach (RoBERTa). These systems were rigorously trained and evaluated across multilabel, multiclass, and binary settings, revealing the superior performance of the RoBERTa model over XGBoost. While both models displayed commendable results, the RoBERTa model consistently demonstrated better performance in classifying prostate cancer findings across all problem formulations.

A significant contribution of our study lies in filling a crucial gap in the literature by focusing on Spanish-language radiological MRI prostate reports. This unique aspect adds depth to the understanding and applicability of automated text classification systems in diverse linguistic contexts. The training and evaluation of models on Spanish reports offer valuable insights for healthcare professionals and researchers dealing with similar datasets.

Moreover, our successful integration of the best-performing multiclass system into the radiological clinic’s information systems as an API marks a crucial milestone. This implementation ensures seamless accessibility and utilization of the developed system by medical professionals, significantly enhancing their diagnostic efficiency and accuracy in managing prostate cancer.

Overall, our study highlights the effectiveness of automated text classification systems in the field of prostate cancer diagnosis, with the RoBERTa model demonstrating superior performance. This research contributes to the ongoing efforts in leveraging machine learning and natural language processing techniques for accurate and efficient cancer diagnosis, ultimately aiding in improved patient outcomes.
